# Discovering Pair-wise Synergies in Microarray Data

**DOI:** 10.1038/srep30672

**Published:** 2016-07-29

**Authors:** Yuan Chen, Dan Cao, Jun Gao, Zheming Yuan

**Affiliations:** 1 Hunan Provincial Key Laboratory for Biology and Control of Plant Diseases and Insect Pests, Hunan Agricultural University, Changsha, Hunan, 410128, China; 2 Hunan Provincial Key Laboratory for Germplasm Innovation and Utilization of Crop, Hunan Agricultural University, Changsha, Hunan, 410128, China; 3 Orient Science &Technology College of Hunan Agricultural University, Changsha, Hunan, 410128, China; 4 College of Resources & Environment, Hunan Agricultural University, Changsha, Hunan, 410128, China; 5 Department of Biochemistry and Molecular Biology, University of Arkansas for Medical Sciences, Little Rock, Arkansas, 72205, USA

## Abstract

Informative gene selection can have important implications for the improvement of cancer diagnosis and the identification of new drug targets. Individual-gene-ranking methods ignore interactions between genes. Furthermore, popular pair-wise gene evaluation methods, *e.g*. TSP and TSG, are helpless for discovering pair-wise interactions. Several efforts to discover pair-wise synergy have been made based on the information approach, such as EMBP and FeatKNN. However, the methods which are employed to estimate mutual information, *e.g*. binarization, histogram-based and KNN estimators, depend on known data or domain characteristics. Recently, Reshef *et al*. proposed a novel maximal information coefficient (MIC) measure to capture a wide range of associations between two variables that has the property of generality. An extension from *MIC*(*X*; *Y*) to *MIC*(*X*_1_; *X*_2_; *Y*) is therefore desired. We developed an approximation algorithm for estimating *MIC*(*X*_1_; *X*_2_; *Y*) where *Y* is a discrete variable. *MIC*(*X*_1_; *X*_2_; *Y*) is employed to detect pair-wise synergy in simulation and cancer microarray data. The results indicate that *MIC*(*X*_1_; *X*_2_; *Y*) also has the property of generality. It can discover synergic genes that are undetectable by reference feature selection methods such as *MIC*(*X*; *Y*) and TSG. Synergic genes can distinguish different phenotypes. Finally, the biological relevance of these synergic genes is validated with GO annotation and OUgene database.

Cancer tissue sample microarray expression data typically possess a common property—the number of samples is much smaller than the number of features—here those features are genes^1^. Informative gene selection has important implications for the improvement of cancer diagnosis, the selection of targeted therapeutics, and the identification of new drug targets^2^,^3^. Individual-gene-ranking methods, such as the *t* test for binary class differentiation^4^ and the *F* test for multi-class differentiation rank genes by comparing the expression values of the same individual gene between different classes. Although these individual-gene methods may discover individual effect genes efficiently, they may have ignored interactions (*i.e*., redundancy and synergy) between genes^4^,^5^,^6^. The interactions between genes are critical in pathway dysregulations which trigger carcinogenesis^7^. Table 1 illustrates an example case of synergy between Gene *X*_1_ and Gene *X*_2_: 1) Knowledge regarding the state of only one of these two variables leaves the state of *Y* uncertain. 2) When states of both *X*_1_ and *X*_2_ are known, then the state of *Y* becomes certain.

Pair-wise gene evaluation has been implemented in several popular algorithms, including top scoring pair (TSP)^8^,^9^, top scoring genes (TSG)^2^, and doublets (*sum*, *diff*, *mul* and *sign*)^7^, which all compare expression values of the same sample between two different genes. However, these methods are incapable of discovering pair-wise interactions efficiently. For example, let *X*_1_ and *X*_2_ be two independent random variables; *Y* equals |*X*_1_–*X*_2_| and is binarized with a median (Fig. 1). Then, the Δ-score for TSP is 0.04, the *χ*^2^-score for TSG is 0.18, and the *t*-score is 0.04, 0.18, 3.42, and 0.56 for *sum*, *diff*, *mul*, and *sign*, respectively. The synergic pairs, *X*_1_ and *X*_2_, cannot be highlighted with these low scores calculated by these methods.

Based on information theory, the measure of *I*(*X*_1_; *X*_2_; *Y*)^10^,^11^ can be used to identify pair-wise interactions^12^,^13^,^14^. The interaction of a gene pair with respect to cancer is defined as


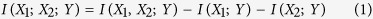


Where *I* is the symbol for mutual information (MI), *X*_1_ and *X*_2_ are random variables representing the expression levels of the two genes and *Y* is a binary random variable representing the presence or absence of cancer^15^. A positive value of *I*(*X*_1_; *X*_2_; *Y*) indicates synergistic interactions, while a negative value of *I*(*X*_1_; *X*_2_; *Y*) indicates redundant interactions.

Several efforts have recently been made to discover pair-wise synergy even multivariate synergy among interacting genes on experimental biological data. The Anastassiou group proposed a systems-based approach called Entropy Minimization and Boolean Parsimony (EMBP) to identify modules of genes that are jointly associated with a phenotype from gene expression data^15^ and SNP data^16^. Anastassiou^11^ emphasized the significance of multivariate analysis such as EMBP for molecular systems biology and clarified the fundamental concepts by explaining the precise physical meaning. Watkinson *et al*.^17^ presented a novel dendrogram-based technique to identify synergies of pairwise genes. Hanczar *et al*.^18^ devised a histogram-based method called FeatKNN to detect the joint effect *I*(*X*_1_, *X*_2_; *Y*). Park *et al*.^19^ proposed a new approach for inferring combinatorial Boolean rules of gene sets for cancer classification by using a synergy network. Shiraishi *et al*.^20^ presented a rank-based non-parametric statistical test for measuring synergistic combinations between two gene sets. Ignac *et al*.^21^ used interaction distances (ID) to identify the most synergic pairs of markers such as SNPs.

Binarization of continuous expression data simplifies the estimation of MI and provides simple logical functions connecting the genes within the found modules^2^,^15^. However, there are multitype complicated patterns in both real-world data (Fig. 2A,B) and simulation data (Fig. 2C,D); binarization might lead to loss of information^11^,^21^. For example, the *IGLC1* gene for the prostate dataset must be trinarized, rather than binarized (Fig. 2C). Several methods have been proposed for the MI estimation, such as kernel density estimation^22^, histogram-based technique^23^, *k*-nearest-neighbor estimator^24^, B-spline functions^25^, Edgeworth^26^, adaptive partitioning^27^,^28^ and dendrogram-based method^17^. Khan *et al*.^29^ evaluated the relative performance of several MI estimation methods, and suggested that the most suitable estimation procedure would depend on known data or domain characteristics and exploratory data analysis. Recently, Reshef *et al*.^30^ presented a novel estimator for two variables called maximal information coefficient (MIC). MIC explores various binning strategies with different numbers of bins, and can capture a wide range of associations, both functional and non-functional, regardless of linear or non-linear relationships. Due to its generality, MIC is becoming widely accepted in scientific research fields^31^. Therefore, there is a large demand for extending MIC from two variables to three variables, even multivariate, to capture a wide range of synergistic interactions^32^.

In this paper, we first developed and described an algorithm to compute *MIC*(*X*_1_; *X*_2_; *Y*). We demonstrated the generality of *MIC*(*X*_1_; *X*_2_; *Y*) with simulation data. We identified the most synergic pairs of genes (not discovered by popular feature selection approaches) using *MIC*(*X*_1_; *X*_2_; *Y*) with several real-world, cancer gene expression profile datasets. Finally, we validated these synergic genes using classification performance, Gene Ontology annotation (GO), and the OUgene database^33^.

## Calculation of *MIC*(*X*_1_; *X*_2_; *Y*) where *Y* is a discrete variable

### Preliminary

Given a finite set *D*_*n* × 3_ = {(x_1_, x_2_, y)| x_1_ ∈ *X*_1_, x_2_ ∈ *X*_2_, y ∈ *Y*}, where *n* is the sample size, *X*_1_ and *X*_2_ are two continuous independent variables, *Y* is the discrete dependent variable *Y* = {*class*_1_, *class*_2_,..., *class*_*P*_}, and *P* is the number of classes, we can partition *X*_1_, *X*_2_, and *Y* into *x*_1_ bins, *x*_2_ bins, and *y* bins, respectively. Here, *y* is fixed as *P*, because *Y* is a discrete variable. We denote such a partition *x*_1_-by-*x*_2_-by-*y* as grid *G*, and the distribution of the data points in *D* on the cells of *G* as *D*^|^_*G*_.

***Definition 1*** For a finite set 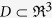 and positive integers *x*_1_, *x*_2_, *y*, define


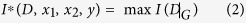


where the maximum is over all grids *G* with *x*_1_-by-*x*_2_-by-*y*, and *I*(*D*|_*G*_) is the *interaction* defined in formula (1).

***Definition 2*** The *characteristic matrix M*(*D*) of a set *D* of three-variable data is an infinite matrix with entries


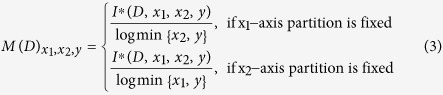


***Definitions 3*** The *maximal interaction coefficient MIC*(*X*_1_; *X*_2_; *Y*) of a set *D* of three-variable data with sample size *n* and grid size less than *B*(*n*) is defined as


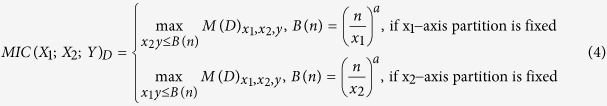


In this paper *a* equals 0.6, the default setting suggested by Reshef *et al*.^30^.

### The maximal grid size *B*(*n*) and normalization of *MIC*(*X*_1_; *X*_2_; *Y*)

Formula (1) can be rewritten as


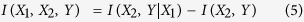



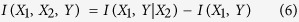


Here *I*(*X*_2_, *Y*|*X*_1_) and *I*(*X*_1_, *Y*|*X*_2_)are conditional mutual information.

According to formula (5) and knowing that the *X*_1_, x_1_-axis partition is fixed, *i.e.* that *X*_1_ is equipartitioned with *x*_1_ bins, the set *D* of three-variable data with sample size *n* can be subdivided into *x*_1_ subsets, and each subset has only two-variable (*X*_2_ and *Y*) and *n*/*x*_1_ samples. The mutual information for each subset can be normalized with log(min{*x*_2_, *y*}) and the maximal grid size *B*(*n*) for each subset should be (*n*/*x*_1_)^*a*^. Therefore, for set *D*, while the x_1_-axis partition is fixed, the normalization benchmark and *B*(*n*) are log (min{*x*_2_, *y*}) and (*n*/*x*_1_)^*a*^, respectively.

Similarly, for set *D* where the x_2_-axis partition is fixed, the normalization benchmark and *B*(*n*) are log (min{*x*_1_, *y*}) and (*n*/*x*_2_)^*a*^, respectively.

### Approximation algorithm for *MIC*(*X*_1_; *X*_2_; *Y*)

Here, we describe the heuristic algorithm, ApproxCharateristicMatrix_3D, for approximating the optimal *MIC*(*X*_1_; *X*_2_; *Y*). It includes four sub-algorithms: EquipartitionX1Axis, SortInIncreasingOrderByX2Value, GetSuperclumpsPartition_3D, and ApproxOptimizeX2Axis. In the dataset *D*, the first and second columns represent *X*_*1*_ and *X*_2_ respectively; the last column represents *Y*. *n* is the sample size. *B* defines the maximal grid size. The symbol “⊥” represents the dataset which is changed from (*a*_1_, *b*_1_, *z*_1_) to (*b*_1_, *a*_1_, *z*_1_). *c* represents the candidate partition point for x-axis. “log” is base-2 logarithm. x_fix_, representing the corresponding x-axis partition, is fixed (x_fix_ ∈ {x_1_, x_2_}). The symbol “ ← ” is an assignment operator.


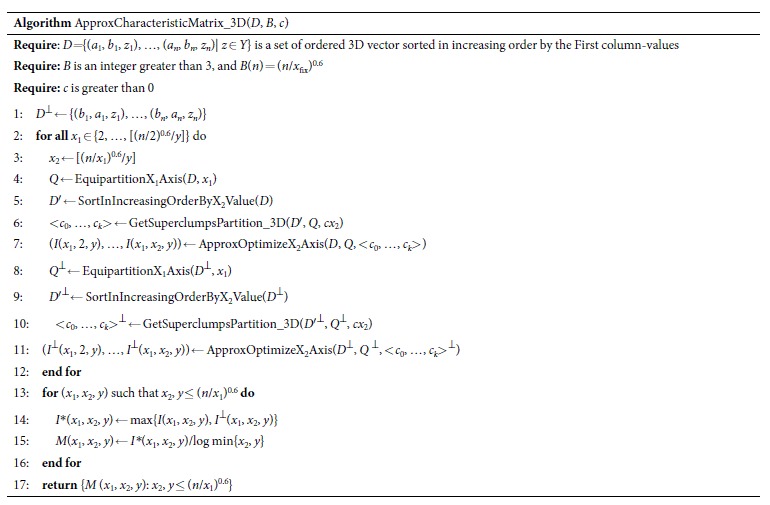



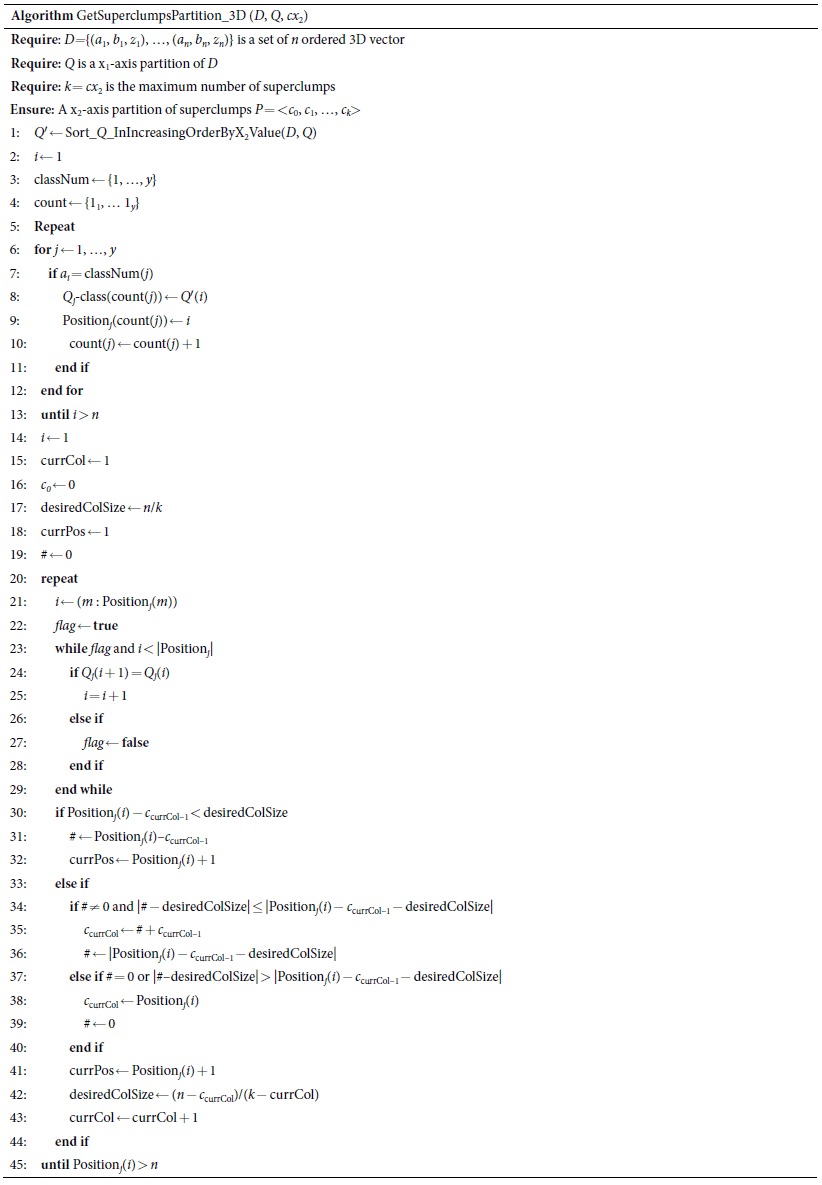



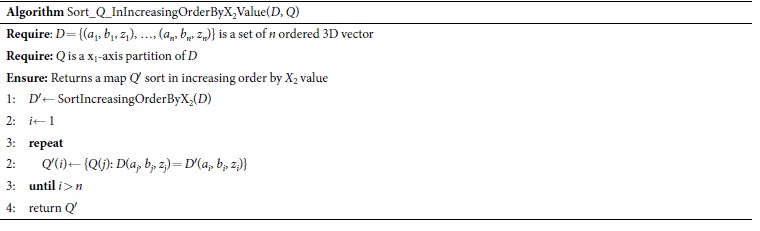


**Figure 3:**
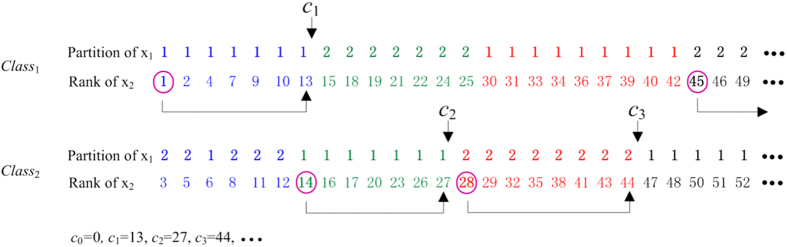
Schematic of getting superclumps partition for three variables. The points with the same color belong to the same superclump.

EquipartitionX1Axis, SortInIncreasingOrderByX2Value and ApproxOptimizeX2Axis are nearly the same as EquipartitionYAxis, SortInIncreasingOrderByXValue, and ApproxOptimizeXAxis in Reshef *et al*.^30^, respectively, except that ApproxOptimizeX2Axis uses *I*(*X*_1_; *X*_2_; *Y*) in place of *I*(*X*; *Y*). Here we demonstrate an example of a superclumps partition (see Fig. 3) and list only the pseudo-code of GetSuperclumpsPartition_3D, which is our core algorithm for calculating interactions. The algorithm includes three steps: 1) divide the data into *P* parts according to *Y*; 2) fix an equipartition of size *x*_1_ on x_1_-axis; and 3) ensure points in the same superclump to be a unit in the same *class*, with the rank of x_2_-axis.

## Results

### Generality of *MIC*(*X*_1_; *X*_2_; *Y*) according to simulation analysis

If *X*_1_ and *X*_2_ are statistically independent of *Y*, *MIC*(*X*_1_; *X*_2_; *Y*) should be close to 0. For example, let *X* and *Y* be two independent, random variables and *Y* is binarized with a median (sample size *n* = 200 and 500 replicates), then *MIC*(*X*; *Y*) = 0.1702 ± 0.0292. Similarly, let *X*_1_, *X*_2_ and *Y* be three independent, random variables, then *MIC*(*X*_1_; *X*_2_; *Y*) = 0.1562 ± 0.0230. *MIC*(*X*_1_; *X*_2_; *Y*) is reasonable in scope compared with *MIC*(*X*; *Y*), and decreases as the sample size grows (0.0596 ± 0.0012, *n* = 20000) and finally converges to 0.

**Figure 4:**
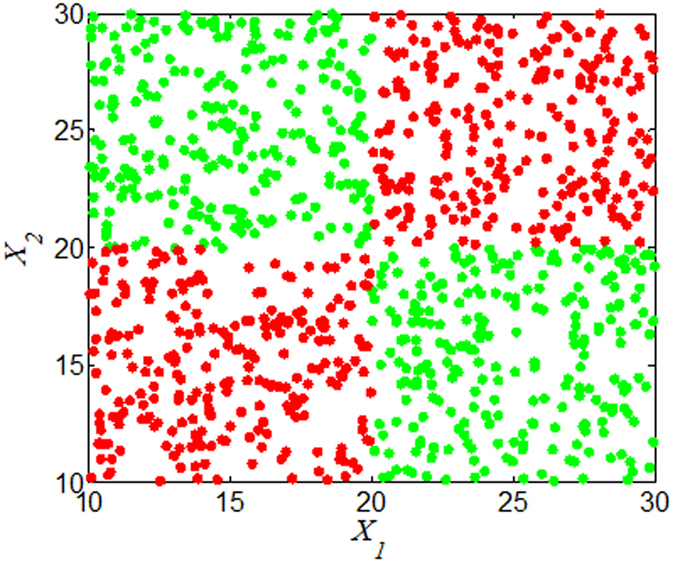
*Y* completely determined by the *synergy* between *X*_1_ and *X*_2_. *X*_1_ and *X*_2_∈[10, 30], 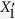 and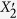 result from binarization vector of *X*_1_ and *X*_2_, respectively. *Y* = 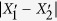
(*n* = 1000). Green and red dots represent *Y* = 1 and *Y* = 0, respectively.

If the state of *Y* is completely determined by the *synergy* between *X*_1_ and *X*_2_, then *MIC*(*X*_1_; *X*_2_; *Y*) should be 1, and *MIC*(*X*; *Y*) should be close to 0. As shown in Fig. 4, *MIC*(*X*_1_; *X*_2_; *Y*) = 1, *MIC*(*X*_1_;*Y*)  = 0.0379 and *MIC*(*X*_2_; *Y*) = 0.0533. If *Y* is a noiseless function of *X*_1_ and *X*_2_, and *X*_1_ is fully redundant of *X*_2_, then *MIC*(*X*_1_; *X*_2_; *Y*) should be −1. For example, 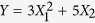 and *X*_1_ = *X*_2_, *MIC*(*X*_1_; *X*_2_; *Y*) = −1, *MIC*(*X*_1_;*Y*) = 1 and *MIC*(*X*_2_;*Y*) = 1.

**Figure 5:**
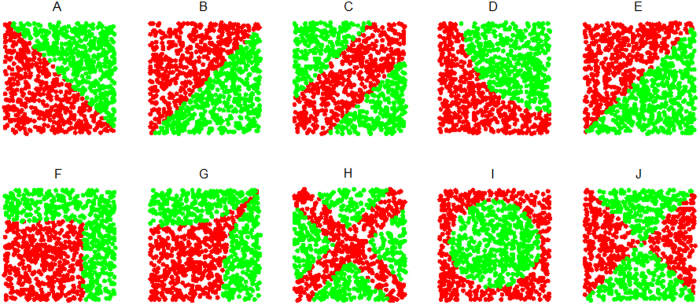
Ten noiseless functions with *Y* = *f* (*X*_1_, *X*_2_). *Y* is binarized with median, green and red dots represent *Y*=1 and *Y*=0, respectively.

If *Y* is a noiseless function of *X*_1_ and X_2_, then the joint effect, *i.e.*, the sum of *MIC*(*X*_1_; *X*_2_; *Y*), *MIC*(*X*_1_; *Y*) and *MIC*(*X*_2_; *Y*), should be 1. Scores of the three components and the joint effect for 10 noiseless functions (Fig. 5) are listed in Table 2. All of the joint effects are close to 1 (0.9672~1.1675). This indicates that the value of *MIC*(*X*_1_; *X*_2_; *Y*) calculated with ApproxCharateristicMatrix_3D is credible, while the value of *MIC*(*X*; *Y*) calculated with ApproxMaxMI^30^ has been widely accepted. From all of the above, we deduce that *MIC*(*X*_1_; *X*_2_; *Y*) can capture a wide range of *interactions*, not limited to specific function types. That is, *MIC*(*X*_1_; *X*_2_; *Y*) has the property of generality.

### Informative genes of synergy pairs discovered by *MIC*(*X*_1_; *X*_2_; *Y*)

We employ *MIC*(*X*_1_; *X*_2_; *Y*) to detect pair-wise synergic genes in three real-world datasets. The literature resources, sample size, number of genes, and the number samples of each class in each dataset are summarized in Table 3.

Four popular gene selection methods, including *MIC*(*X*; *Y*), minimum-redundancy maximum-relevancy (mRMR)^34^, support vector machine recursive feature elimination (SVM-RFE)^35^,^36^ and TSG^2^, are chosen to compare with *MIC*(*X*_1_; *X*_2_; *Y*). The *MIC*(*X*; *Y*) estimator (setting *a* = 0.6 and *c* = 5) of Reshef *et al*.^30^ is available at http://www.exploredata.net/, MIQ-MRMR is available at http://home.penglab.com/, and an *R* Package implementation of SVM-RFE is available at http://www.uccor.edu.ar/paginas/seminarios/software/SVM-RFE.zip. The TSG algorithm from our previous report^2^ is available upon request.

**Figure 6:**
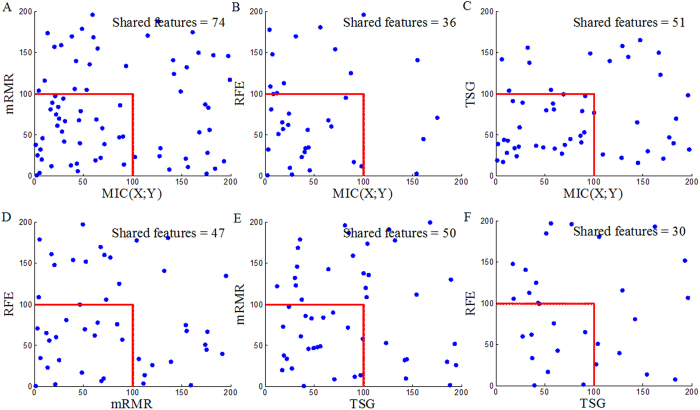
Overlaps among the Top200s selected by *MIC*(*X*; *Y*), MRMR, SVM-RFE and TSG in the Prostate dataset.

**Figure 7:**
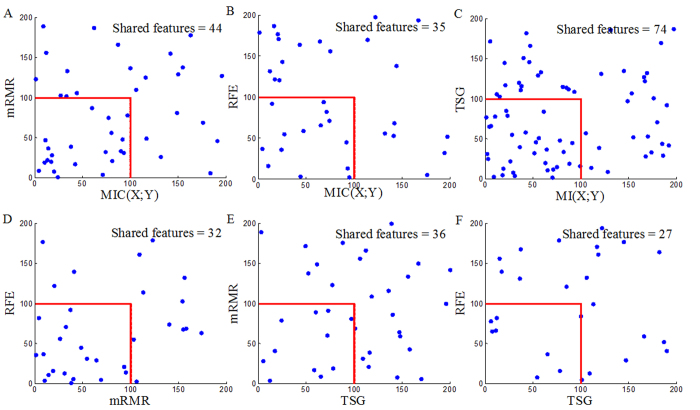
Overlaps among the Top200s selected by *MIC*(*X*; *Y*), MRMR, SVM-RFE and TSG in the DLBCL dataset.

**Figure 8:**
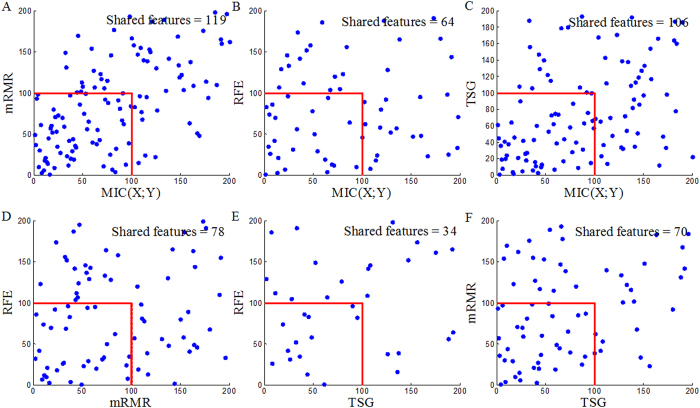
Overlaps among the Top200s selected by *MIC*(*X*; *Y*), MRMR, SVM-RFE and TSG in the Lung dataset.

Each reference method ranks the top 200 genes (Top200s) for each dataset (Top200s are shown in the Supplementary Material Table S1-S3). The Top200s identified by different reference methods are compared with each other. We can observe significant overlaps between the Top 200s selected by the four reference methods, as shown in Figs 6, 7 and 8. This indicates that a considerable number of similar informative genes can be detected by these reference methods. *MIC*(*X*; *Y*) is an individual-gene-filter method and can only highlight genes that are individually discriminant. Although mRMR, SVM-RFE and TSG are not individual-gene-filter methods; the Top200s selected by them have considerable similarities to the Top200s selected by *MIC*(*X*; *Y*). This indicates that these methods can efficiently discover genes that are individually discriminant, but not specific to the genes have pair-wise synergy effects.

**Figure 9:**
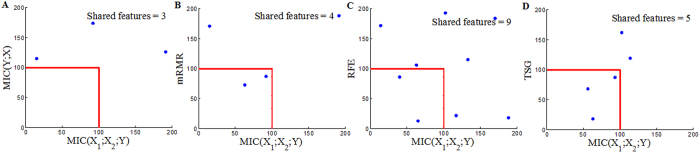
Overlaps between the Top200 selected by *MIC*(*X*_1_; *X*_2_; *Y*) and the Top200s selected by *MIC*(*X*; *Y*), MRMR, SVM-RFE and TSG in the Prostate dataset.

**Figure 10:**
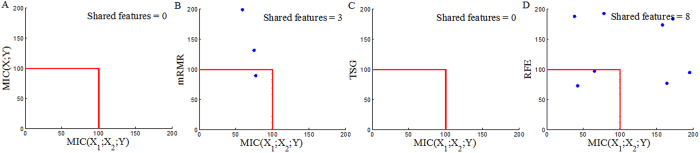
Overlaps between the Top200 selected by *MIC*(*X*_1_; *X*_2_; *Y*) and the Top200s selected by *MIC*(*X*; *Y*), MRMR, SVM-RFE and TSG in the DLBCL dataset.

**Figure 11:**
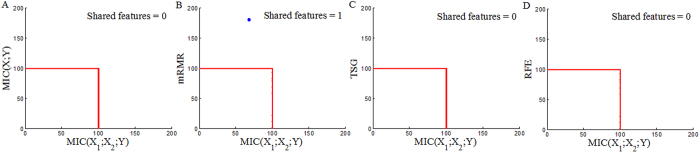
Overlaps between the Top200 selected by *MIC*(*X*_1_; *X*_2_; *Y*) and the Top200s selected by *MIC*(*X*; *Y*), MRMR, SVM-RFE and TSG in the Lung dataset.

Now, we employ *MIC*(*X*_1_; *X*_2_; *Y*) to detect pair-wise synergic genes. *MIC*(*X*_1_; *X*_2_; *Y*) ranks the top 117, 117 and 110 pair-wise genes for Prostate, DLBCL and Lung1, respectively. After removing repeated genes, we obtain three Top200s (Top200s are shown in the Supplementary Material Table S1–S3). We compare our *MIC*(*X*_1_; *X*_2_; *Y*) results with the results from four above mentioned reference selection methods. Clearly, the Top200s selected by *MIC*(*X*_1_; *X*_2_; *Y*) has little overlap with the Top200s selected by the others (Figs 9, 10 and 11). We, therefore, deduce that *MIC*(*X*_1_; *X*_2_; *Y*) can discover new synergic genes and that the other four reference feature selection methods can only discover genes that are individually discriminant.

### Synergic gene justification

**Figure 12:**
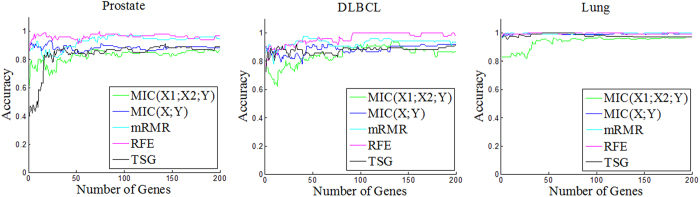
Prediction accuracy of five feature selection methods combined with SVC Classifier over three datasets.

We initially validate these synergic genes according to their prediction performance with a supported vector classifier (SVC). SVC is available at http://prtools.org/ software/. Fig. 12, illustrates the 10-fold cross-validation prediction accuracies using genes from Top1 to the Top200 selected by *MIC*(*X*_1_; *X*_2_; *Y*), as well as by *MIC*(*X*; *Y*), MRMR, SVM-RFE and TSG. *MIC*(*X*_1_; *X*_2_; *Y*) receives comparable accuracies. This indicates that these synergic genes have sufficient ability to distinguish tissue and cancer types, from the perspective of machine learning.

Do the synergic genes selected by *MIC*(*X*_1_; *X*_2_; *Y*) have any biological relevance to tissue or cancer type? This is particularly relevant considering that even a random set of genes may be a good predictor of cancer sample definition^37^. Therefore, we further validated these synergic genes, using the Prostate dataset as an example, according to GO annotation and OUgene database.

**Figure 13:**
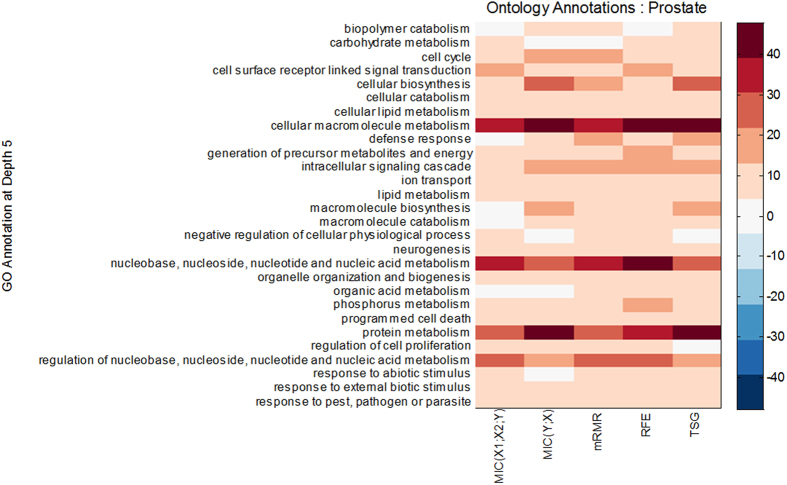
GO annotations for the Top200s selected by different methods in the Prostate dataset. Deeper colors of one point in the figure means the terms covered with more genes. We have removed the terms in which the sum of genes number is less than 25 across all methods.

We used the GATHER system^38^ (http://gather.genome.duke.edu/) to query GO annotations associated with the Top200s selected by the five methods, as shown in Fig. 13. Although there is little overlap between the genes selected by *MIC*(*X*_1_; *X*_2_; *Y*) and the genes selected by the four reference methods (Figs 9, 10 and 11), synergic genes share the same four heavily marked terms with genes that are individually discriminant (Fig. 13). These four heavily marked GO terms are “cellular macromolecule metabolism,” “nucleobase, nucleoside, nucleotide and nucleic acid metabolism,” “protein metabolism,” and “regulation of nucleobase, nucleoside, nucleotide and nucleic acid metabolism”.

The current version of OUgene, a disease associated, over-expressed and under-expressed gene database, includes 7,238 gene entries, 1,480 diseases entries, and 56,442 PubMed links. We ranked the Top200 synergic genes out of the 12,600 genes in the Prostate dataset using *MIC*(*X*_1_; *X*_2_; *Y*). Of these Top200, 67 tumorigenesis genes were queried against OUgene, and 18 of them have been reported related to prostate cancer^39^,^40^,^41^,^42^,^43^,^44^,^45^,^46^,^47^,^48^,^49^,^50^,^51^,^52^,^53^,^54^,^55^,^56^ (Table 4).

### Combined synergic and individual effect genes to improve the prediction performance

The MicroArray Quality Control (MAQC)-II project provided benchmark datasets for the development and validation of microarray-based predictive models^57^. We use the Breast Cancer dataset from MAQC-II to further evaluate the reliability of *MIC*(*X*_1_; *X*_2_; *Y*). This dataset is used to predict the pre-operative treatment response (pCR) and estrogen receptor status (erpos). It was originally grouped into two groups: a training set containing 130 samples (33 positivesand 97 negatives for pCR, 80 positives and 50 negatives for erpos), and a validation set containing 100 samples (15 positives and 85 negatives for pCR, 61 positives and 39 negatives for erps). Raw probe data (CEL files) for a set of Affymetrix Human Genome U133A Array microarray assays were obtained from GEO (GSE20194). The microarray chip had probe sets for 22283 features, which were normalized and summarized using the Robust Multi-array Average (RMA) method^58^ on perfect match probes only. Sequential forward selection (SFS) is used to select individually discriminant genes and synergic genes with MIC(*X*; *Y*) and MIC(*X*_1_; *X*_2_; *Y*), respectively: (i) Rank the genes separately by *MIC*(*X*; *Y*) or *MIC*(*X*_1_; *X*_2_; *Y*); (ii) select the Top200 genes (Listed in supplemental material Table S4–S7), and conduct 10-fold cross-validation (CV10) for the training sets based on SVC. Accuracy was denoted as CV10_*w*_ (*w* = 1, …. 200); (iii) the genes with the highest CV10 accuracy were selected as informative genes for validation. We use the accuracy and Matthew correlation coefficient (*MCC*) to evaluate the predictive power of the analysis.


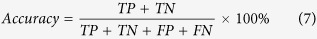



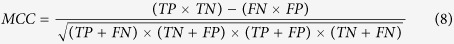


Here *TP*, *TN*, *FP*, *FN* denote true positives, true negatives, false positives and false negatives respectively. Greater accuracy and *MCC* represent better prediction ability of a model.

As shown in Table 5, for Breast erops, the accuracies of individual model and synergic model are 89% and 90%, the *MCC*s are 0.77 and 0.79, respectively. If we integrate the two models, the accuracy and *MCC* of combined model are improved into 92% and 0.83, respectively (Better results may be achieved while the redundancies among genes are removed). Similar improved effects are observed in the “Breast pCR” dataset analysis. These results demonstrate that synergic genes selected by *MIC*(*X*_1_; *X*_2_; *Y*) enhance the individually discriminant model for improving prediction performance.

## Discussion

**Figure 14:**
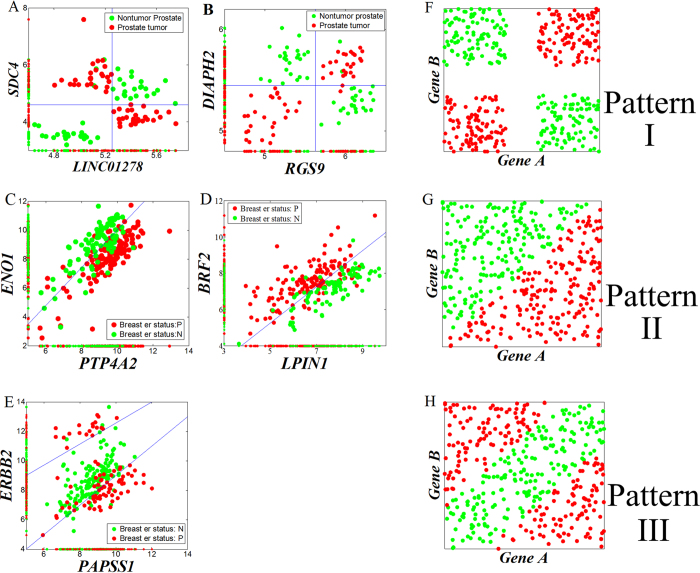
Three representative patterns of pair-wise synergy identified by *MIC*(*X*_1_, *X*_2_: *Y*) method. (**A–E)** are from real-world datasets, (**F–H**) are the corresponding hypothetical extreme examples.

We scanned the Top200s genes selected by *MIC*(*X*_1_; *X*_2_; *Y*) on Prostate and Breast cancer datasets, and summarized three representative patterns of pair-wise synergy and their corresponding theoretic distribution (Fig. 14). Pattern I (Fig. 14A,B,F) results from the typical synergy of Fig. 4, Pattern II (Fig. 14C,D,G) results from the function *y* = *x*_1_–*x*_2_ (Fig. 5B), and Pattern III (Fig. 14E,H) results from the function *y* = |*x*_1_ *–* *x*_2_| (Fig. 5C). These patterns offer an efficient tool to infer pathogenic mechanism, even to provide a quantitative model, of pair-wise synergy genes. For Pattern I, *Gene A* and *Gene B* both could be on-off oncogenes (Fig. 14A) or tumor suppressor genes (Fig. 14B) which inhibit each other. For Pattern II, one could be an oncogene, and the other could be a tumor suppressor gene. Pattern III is similar to Pattern I, but *Gene A* and *Gene B* both could be non on-off oncogenes. The results indicate that although the synergy pattern is diversified in real-world datasets, the *MIC*(*X*_1_; *X*_2_; *Y*) method can explore them well. For the pair-wise synergy *ERBB2*-*PAPSS1*, they have been widely reported to correlate with breast cancer^59^,^60^,^61^,^62^, as well as the *ENO1- PTP4A2* pair^63^,^64^,^65^,^66^. For the *BRF2*-*LIPIN1* pair, *BRF2* is related to tumor angiogenesis^67^. *LIPIN1* has been reported to correlate with non-tumorous diseases such as rhabdomyolysis^68^, Type 2 diabetes^69^, metabolic syndrome^70^ and acute myoglobinuria^71^. Recently, *LIPIN1* was reported to regulate breast adenocarcinoma cell proliferation rate^72^. For the *SDC4-LINC01278* pair, *SDC4* has been reported to correlate with tumors^73^, but *LINC01278* has not. For the *RGS9*-*DIAPH2* pair, neither of them has been reported to correlate with cancer. However, *MIC*(*X*_1_; *X*_2_; *Y*) suggests that *LINC01278*, *RGS9* and *DIAPH2* are important informative genes for prostate tumors, and should be given proper attention.

“MIC is a great step forward, but there are many more steps to take”^32^. In this article we took such a step—the extension of two variables to three variables which consider pair-wise interaction. Based on “exploring various binning strategies with different number of bins”, Reshef *et al*.^30^ employed a clump (points in the same clump to be a unit) partition technique to reduce computing time and improve estimation accuracy of MI in a two-dimensional space. This technique does not work in a three-dimensional space, because the definition of clump/superclump has changed. We re-defined superclumps as “points in the same superclump to be a unit in the same *class*, with the rank of x_2_-axis” for considering three variables as a whole, and designed a novel algorithm illustrated in Fig. 3 to overcome this barrier. However, complicated diseases such as cancer are often related to collaborative effects involving interactions of multiple genes. Multivariate analysis, just as Anastassiou group^11^,^15^,^16^,^17^, Park *et al*.^19^ and Shiraishi *et al*.^20^ did, is going to be the trend. An extension from *MIC*(*X*_1_; *X*_2_; *Y*) to MIC-based multivariate association networks is therefore still desire.

## Additional Information

**How to cite this article**: Chen, Y. *et al*. Discovering Pair-wise Synergies in Microarray Data. *Sci. Rep.* **6**, 30672; doi: 10.1038/srep30672 (2016).

## Supplementary Material

Supplementary Information

## Figures and Tables

**Figure 1:**
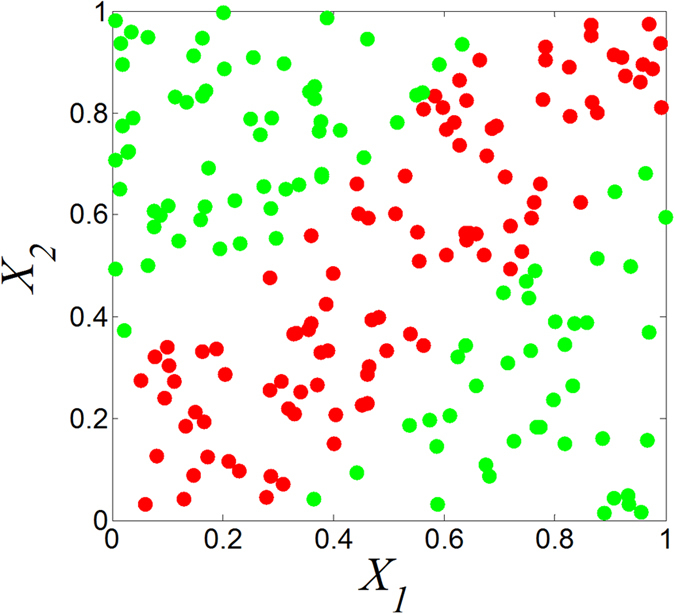
Synergic pairs conducted by function. *Y* = |*X*1 – *X*2|(*n* = 200). *Y* is binarized with a median. Red point: positive sample. Green point: negative sample.

**Figure 2:**
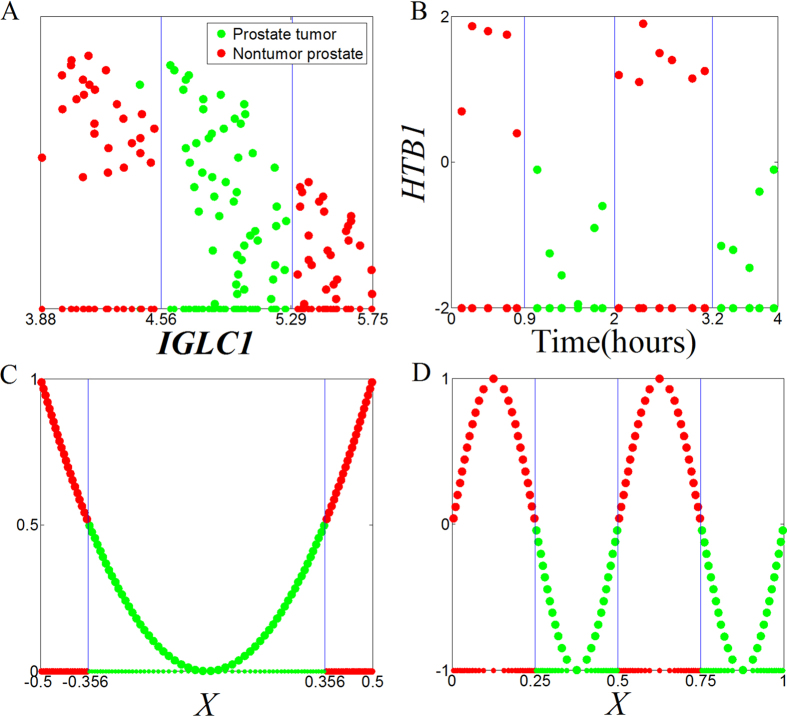
Examples of scatter plots of discretization for gene expression. (**A,B**) are real-word gene expression values for prostate dataset^74^ and yeast dataset^75^; the values of *HTB1* gene are binarized with 0. C and D are simulation datasets from *Y* = 4·*X*^2^ and *Y* = sin (4·π·*X*), *Y* is binarized with 0.5 and 0, respectively. Red point: positive sample. Green point: negative sample.

**Table 1:** A typical pair-wise synergy between *X*_1_ and *X*_2_.

*Y*	*X*_1_	*X*_2_	*X*_1_ ⊕ *X*_2_
−	1	1	0
−	0	0	0
+	1	0	1
+	0	1	1

⊕ is an exclusive-or operation.

**Table 2:** Mean scores of the three components and the joint effect for 10 noiseless functions (*n* = 1000, 1000 replicates).

Function	Domain of *X*_1_	Domain of *X*_2_	*Y* = *f*(*X*_1_, *X*_2_)	*MIC*(*X*_1_; *X*_2_; *Y*)	*MIC*(*X*_1_; *Y*)	*MIC*(*X*_2_; *Y*)	Joint effect
A	[0, 1]	[0, 1]	x_1_+x_2_	0.3667	0.3817	0.3798	1.1283
B	[0, 1]	[0, 1]	x_1_*−*x_2_	0.3793	0.3824	0.3663	1.1280
C	[0, 1]	[0, 1]	ABS(x_1_−x_2_)	0.8222	0.1287	0.1281	1.0790
D	[0, 1]	[0, 1]	x_1_×x_2_	0.3215	0.4134	0.4144	1.1493
E	[0, 1]	[0, 1]	x_1_/x_2_	0.3835	0.3804	0.3653	1.1292
F	[5, 23.3]	[5, 23.3]	10^x^_1_+10^x^_2_	0.2390	0.4657	0.4628	1.1675
G	[0, 1]	[0, 1]	ABS(1000^x^_1_−1000^x^_2_)	0.4555	0.3386	0.3381	1.1322
H	[0, 1]	[0, 1]	ABS(ABS(x_1_−0.5)−ABS(x_2_−0.5))	0.7080	0.1295	0.1298	0.9672
I	[0, 3.13]	[1.5, 4.75]	LOG_2_(ABS(SIN(x_1_)−COS(x_2_)))	0.2853	0.3824	0.4274	1.0950
J	[0, 3]	[0, 3]	SIN(x_1_)−SIN(x_2_)	0.3044	0.3848	0.3832	1.0723

**Table 3:** Three binary-class gene expression datasets.

Dataset	No. of Genes	No. of samples	No. of samples in class I	No. of samples in class II	Reference
Prostate	12600	102	52	50	74
Lung	12533	181	150	31	76
DLBCL	7129	77	58	19	77

**Table 4:** The 67 cancer related genes out of the Top200 selected by *MIC*(*X*_1_; *X*_2_; *Y*) in the Prostate dataset.

Genes	Related tumors
ABCB1, AMACR, CAV1, CCND1, CSF2, DPT, E2F3, ETV4, GOT2, GREB1, HBP1, HCLS1, HMGA1, PAX2, SFRP1, SOX9, TRAF4, ZNF143	Prostate
ABCA4, CASC3, CD81, COMP, MAP1LC3B, PPP3CA, SLN, TFAP2C, TRO	Breast cancer
DSC2, EDG4, FBLN1, GALNT3, KRT10, NDN	Ovarian carcinomas
CTSE, DNAJA1, LY6E	Pancreatic cancer
NR2F6, TERF2, TPP1	Colorectal cancer
PCBP2, RAF1	Glioma
COL6A1, CYP2A13	Lung cancer
PPP2R5C	leukemia
PPP6C	Hepatocellular carcinoma
AGXT	Lymphomas
DIO2	Thyroid carcinomas
DYRK2	Lung adenocarcinomas
FGFBP1	Gallbladder cancer
PROP1	Pituitary adenoma
PITX3	Liposarcoma
RFP	Oligodendroglioma
CDKN1C	Adrenal adenoma
VAV1	Ovarian carcinomas, Leukemia
JAG1	Breast cancer, Cervical cancer
PHGDH	Breast cancer, Cervical cancer
HYAL1	Breast cancer, Laryngeal carcinoma, Pancreatic cancer
NCAM1	Sarcoidosis, Leukemia, Lymphomas
PPP2R2A	Squamous cell carcinoma, Leukemia, Esophageal cancer, Lung cancer
GATA2	Breast cancer, Leukemia, Neuroblastoma, Choriocarcinoma
THBS2	Breast cancer, Adenocarcinoma, Colorectal cancer, Ovarian carcinomas
WNT5A	Breast cancer, Leukemia, Pancreatic cancer, Ovarian carcinomas, Melanoma
TGM2	Adenocarcinoma, Neuroblastoma, Pancreatic cancer, Ovarian carcinomas, Lung cancer, Hepatocellular carcinoma, Melanoma
GSTP1	Squamous cell carcinoma, Leukemia, Lymphomas, Ovarian carcinomas, Lung cancer, Hepatocellular carcinoma, Melanoma, Colon cancer, Glioblastoma multiforme, Astrocytoma, Osteosarcoma
BAI1	Carcinoma
PTP4A3	Carcinoma
TGFBR3	Carcinoma

**Table 5:** Results of independent test for erpos and pCR of Breast cancer.

Dataset	Model	Number of genes	Validation accuracy	Validation *MCC*
Breast				
erpos	Individual model, genes selected by *MIC*(*X*; *Y*)	8	89%	0.77
	Synergic model, genes selected by *MIC*(*X*_1_; *X*_2_; *Y*)	34	90%	0.79
	Combined model, genes selected by *MIC*(*X*; *Y*) and *MIC*(*X*_1_; *X*_2_; *Y*)	42	92%	0.83
	Candidate model in reference 51	6	87%	0.73
	Best model in reference 51	316	90%	0.79
Breast				
pCR	Individual model, genes selected by *MIC*(*X*; *Y*)	59	82%	0.36
	Synergic model, genes selected by *MIC*(*X*_1_; *X*_2_; *Y*)	32	81%	0.35
	Combined model, genes selected by *MIC*(*X*; *Y*) and *MIC*(*X*_1_; *X*_2_; *Y*)	91	84%	0.37
	Candidate model in reference 51	206	72%	0.30
	Best model in reference 51	40	73%	0.38
