# Supplementary material for: Discovering Pair-wise Synergies in Microarray Data
Source: Sci Rep. 2016 Jul 29;6:30672. doi: 10.1038/srep30672 (PMC4965793; doi:10.1038/srep30672)
Supplement: Supplementary Information [file srep30672-s1.pdf]

Discovering Pair-wise Synergies in Microarray Data

Yuan Chen [1, 2, +], Dan Cao [3, +], Jun Gao [4,5,*] and Zheming Yuan [1, 2,*]

Table S1: Top 200 genes indices selected by different methods from prostate1

	MIC_{Y-2X}	MIC_{Y-X}	mRMR	RFE	TSG
1	2149	10494	6185	6185	143
2	8239	6185	11013	10537	1041
3	2181	9172	6145	10234	2078
4	11934	8965	12153	5045	2532
5	2326	12153	8878	497	2730
6	3043	9850	5890	4438	7264
7	500	4365	7887	3649	7672
8	8539	12148	10553	7539	7986
9	2835	8554	7428	7139	8559
10	8633	10138	12098	205	8933
11	6731	8850	4285	6447	9129
12	7155	6462	9093	10215	10465
13	4554	7067	11942	6220	10670
14	10951	299	10996	11858	12082
15	5262	6620	10956	7875	12483
16	10823	7557	9505	2237	11215
17	9744	9093	6228	8123	4365
18	11170	9593	7775	5632	11871
19	10053	2839	12132	470	10494
20	12062	7247	4365	9178	8986
21	6982	5757	10234	7602	9354
22	11977	288	11818	4636	7905
23	4871	9034	9057	11942	5977
24	6386	3794	2760	8149	5757
25	139	205	9172	7903	7315
26	1264	7756	6944	7623	8527
27	10139	10537	1388	9118	11818
28	11057	8631	8668	2371	8850
29	8198	6865	7930	12495	8199
30	10518	10614	5402	9900	11467
31	7503	3525	2500	9564	5944
32	8443	6866	9850	9172	8975
33	1411	7066	8741	1221	6359
34	4483	9341	7905	4282	2839
35	1611	3524	8258	5890	8058

36	7555	8566	4928	12196	9034
37	7166	11942	8949	5952	4282
38	9170	942	10494	7298	9063
39	3636	3729	6390	7375	6185
40	8444	12495	6615	7896	11202
41	8177	4282	8516	7408	8200
42	3173	9050	10614	1490	4993
43	9242	10956	6556	7465	6462
44	780	5890	9173	12113	12148
45	9910	3649	6680	8498	12594
46	2292	6615	12148	4105	4513
47	891	6406	9442	9816	6930
48	11162	8058	8123	9044	2097
49	6468	1002	3703	1112	9442
50	5221	8011	270	7239	5155
51	5254	6941	6842	7067	8768
52	6560	6824	4243	4666	10112
53	10067	8306	6643	5991	8123
54	6685	5799	8631	11640	4772
55	7796	4690	7147	11263	5492
56	2014	6037	6011	10956	3703
57	9406	2279	1485	9593	2912
58	3533	6470	5989	8371	6108
59	4103	5761	6864	5661	3794
60	5021	6359	8868	11818	238
61	9270	3577	9034	7664	8528
62	6814	12132	1292	9034	6773
63	11871	9937	5241	11648	7465
64	3282	9039	9133	8902	10995
65	6220	10749	9576	9093	266
66	7174	329	12516	4069	8176
67	12266	11818	3525	4525	7735
68	11027	1203	3649	9039	2014
69	5697	5989	9937	9735	9195
70	8727	9063	205	6842	7428
71	5749	6390	5110	6145	10672
72	6306	7520	3570	11367	5821
73	6127	729	11871	9327	8083
74	10634	10550	2895	269	7157
75	6550	11064	288	8594	7185
76	11978	12594	10539	3794	7056
77	5647	11052	4804	5838	10677
78	7176	2797	4515	9133	1378
79	6899	5545	6406	4601	3879

80	3490	6497	5909	280	6941
81	5980	5835	7557	9850	5761
82	7284	11258	6381	5862	11216
83	8287	9174	6930	3765	1322
84	9809	10260	3794	11006	3570
85	954	5566	406	3617	6838
86	2942	9442	8200	8444	7437
87	1663	8200	6438	10732	7292
88	9203	3879	7500	12460	6470
89	7661	1256	9593	8610	10537
90	9453	8123	9195	9157	7405
91	2507	10996	7617	4960	9093
92	6438	8473	7717	10558	5574
93	7292	6765	9422	9403	9626
94	7337	8999	6865	8603	9230
95	8310	8018	6481	11258	6511
96	9486	7884	9267	10578	2443
97	10161	2977	5757	5042	10996
98	3153	10215	6561	8612	8387
99	4534	8589	8085	8368	5989
100	1963	10677	9911	12148	4726
101	9032	590	11315	6462	6512
102	10417	9057	12051	7329	7623
103	3302	8067	12352	6360	4514
104	4409	8277	8965	3741	7067
105	5098	8074	8306	1613	6037
106	11764	9635	4282	11871	8591
107	8293	7821	1806	4905	9215
108	9617	8729	11644	8026	10383
109	3062	8527	4514	12153	8330
110	6719	11240	8367	9331	7952
111	8780	7820	5045	9964	10358
112	7327	9048	11858	4319	7791
113	9000	7469	8970	2839	4723
114	6392	8889	8615	44	12454
115	7766	5262	8851	8981	11854
116	5693	10907	10138	10092	12256
117	4636	7772	3834	7482	10106
118	5887	9176	959	1860	8724
119	6938	12545	283	5524	6392
120	9065	5508	7623	6063	8455
121	711	8952	7943	9410	4417
122	6894	4241	10465	9295	7913
123	9185	12414	5944	10207	6643

124	11805	6119	2791	11403	5684
125	4971	9599	8270	8200	10439
126	11252	10840	7519	7265	7896
127	5882	2760	2985	8943	1761
128	8056	7905	9255	5707	769
129	5831	6237	5401	4751	10092
130	1793	9897	6645	6066	3985
131	876	3768	5792	6688	3780
132	8323	12203	11467	10162	12455
133	8981	6394	4618	9291	7272
134	8236	1219	6765	8818	5283
135	512	996	8651	9470	11073
136	11501	6033	6037	6200	7068
137	1604	10553	8900	3566	2873
138	2625	6933	4726	6024	9341
139	7719	8186	9900	163	6745
140	9330	6995	9050	2764	10907
141	3449	8552	8552	11467	8747
142	4616	2791	11577	9104	9850
143	5587	6266	10995	12600	12098
144	7032	266	4970	9661	8741
145	11016	11215	10979	5629	996
146	6759	12448	8975	8928	8517
147	7261	549	4856	8817	306
148	1131	4833	7588	4365	6301
149	5955	12352	7992	10875	7884
150	1401	7165	8407	3938	7946
151	10594	6659	1958	7505	6575
152	6709	8103	4022	4243	10056
153	10868	8916	4473	8393	7958
154	9511	10234	8594	6390	11858
155	12416	11467	9039	10658	6766
156	8311	4285	4577	2245	6866
157	12053	7532	7247	4804	47
158	4292	3160	3394	6083	6237
159	4388	9412	10537	7181	2191
160	7396	6176	8249	5110	7746
161	8694	8498	6118	6228	8716
162	10128	2681	7550	4380	10417
163	10380	10261	7048	11849	9791
164	6460	4964	9828	6650	2074
165	10698	4805	5954	7198	549
166	3918	7946	8961	3402	8495
167	6980	8407	9604	7304	7624

168	2170	6643	7451	11920	11932
169	6955	7715	6359	9308	9235
170	8499	8953	942	3525	4535
171	12426	9354	5262	4899	721
172	9184	4737	3912	10951	6263
173	10350	1370	3120	3448	6484
174	652	6438	7067	2247	939
175	6646	6145	8498	1212	4042
176	5314	8668	4525	9047	8019
177	8009	6930	4949	12403	9876
178	11334	6011	12455	8965	12378
179	6440	6715	8058	8878	10505
180	9646	7229	1399	7079	6963
181	7319	11202	5091	6037	4648
182	822	4856	8793	7527	8958
183	6218	11350	9770	11345	7539
184	3238	7428	10481	8499	12399
185	9357	8673	7722	8768	5402
186	9381	5418	11756	11791	8043
187	5571	146	6838	7637	10916
188	5632	7765	10840	10292	11013
189	639	6909	7618	11857	6645
190	8814	6690	5291	7982	11593
191	10840	8690	7896	9892	5791
192	6513	9411	10547	7757	6908
193	11326	7775	5029	10417	4243
194	1508	8664	1941	4995	6944
195	5362	8890	9470	9976	10202
196	11407	8387	5761	10677	4905
197	8844	8975	8767	3703	8641
198	10037	12305	5462	11879	8294
199	4425	3834	4278	9671	12280
200	8205	4989	11932	11787	12268

The number means the location of introducing genes in original dataset.

Table S2: Top 200 genes indices selected by different methods from DLBCL.

	MIC_{Y-2X}	MIC_{Y-X}	mRMR	RFE	TSG
1	155	506	1352	2006	5934
2	364	2988	2928	555	7091
3	4814	972	5292	5130	5867
4	6974	4453	87	5648	2137
5	905	4202	4256	699	1055
6	6804	5409	5518	7102	5930

7	3162	1092	6645	2609	2937
8	5890	2043	5054	3791	2100
9	1019	5867	4453	3015	2912
10	4925	5077	5648	4070	1823
11	2671	6179	5595	613	6524
12	5590	2789	578	6055	87
13	3012	5882	4943	4194	582
14	3816	4028	613	4050	5175
15	253	4485	5616	3361	373
16	3699	4292	3414	5077	4183
17	3778	1480	203	2092	1221
18	4969	1055	5198	86	4273
19	2733	582	5077	4971	4339
20	3601	5054	1480	6573	4372
21	687	3535	2392	6730	5280
22	2676	1430	5882	2044	302
23	3200	4024	4008	6575	3684
24	976	1352	5386	1039	5967
25	4426	1670	2308	5183	972
26	1878	302	2128	3215	2421
27	4757	1818	637	74	1412
28	330	486	1055	1665	3736
29	6744	2100	6580	1612	1969
30	889	5994	2064	6770	5594
31	1075	2137	4194	4903	2988
32	1420	4010	2121	4374	1373
33	289	2657	4463	3408	4330
34	464	4580	2614	6168	4680
35	3170	6295	4856	4584	4130
36	5461	605	4354	1352	2901
37	935	3639	4028	4453	2761
38	6323	2306	2006	3119	6023
39	2414	4545	2306	3011	3693
40	4693	6476	7102	3724	605
41	936	6703	1221	4032	1934
42	1834	203	4553	7037	2941
43	5464	1693	5124	4454	3155
44	4283	5130	4156	2482	4970
45	4650	5629	2933	6725	1119
46	2278	104	171	3660	4153
47	6527	6493	6179	4234	4272
48	4287	3398	6725	5871	349
49	6779	356	4340	6601	6716
50	2888	6385	5322	2797	951

51	6059	1373	339	4784	1860
52	217	834	585	6591	7005
53	1102	4153	1439	4459	1016
54	3147	1704	4903	372	3791
55	2948	880	3409	1818	486
56	3218	1860	5998	2128	4812
57	3278	3120	1760	4142	3461
58	6625	5088	6377	3151	203
59	1455	7008	3121	6493	5031
60	1284	6606	5388	779	1200
61	3981	4503	2244	3622	1829
62	2095	1132	6795	801	5819
63	6839	4372	484	1585	5242
64	4803	2761	1612	6570	6250
65	3276	6524	1930	2937	4453
66	4363	3008	1984	6524	5409
67	335	6047	2433	3878	6834
68	5718	1188	942	3818	492
69	1154	3861	699	3053	2493
70	5300	7091	2452	5552	3494
71	648	87	5193	2121	5480
72	5075	47	2401	4230	5388
73	470	3283	2837	6323	3499
74	4201	2121	276	4518	2557
75	2342	373	5935	1177	5136
76	7010	4991	2164	1128	163
77	717	5935	2656	5464	506
78	6410	349	4116	5930	5077
79	745	5637	5967	760	4024
80	4708	5998	4038	3515	6304
81	731	2392	2669	6154	4780
82	2054	4680	1385	87	2309
83	1233	2383	5972	574	1780
84	4885	1166	1959	778	4503
85	2583	562	1163	3903	1430
86	6541	5579	4546	3909	6191
87	891	438	6606	6141	3868
88	1145	4339	5497	5846	1225
89	5272	385	1200	6359	6140
90	2603	4463	1154	4747	1607
91	4868	1119	3499	930	2527
92	5064	6725	4090	4028	4110
93	6391	4194	6730	4435	2319
94	1330	4061	3732	1188	4567

95	3738	555	4050	1995	5336
96	3172	6549	4410	360	4254
97	3706	4116	5505	1455	2669
98	3179	1024	5938	3086	1350
99	3471	4183	3624	6784	640
100	7033	6815	6113	6644	778
101	878	6603	686	3112	699
102	2704	3005	2657	6040	337
103	6512	2883	1818	1201	4485
104	3720	316	412	7036	4033
105	6842	3461	4768	3913	3921
106	5445	5956	5130	2403	2789
107	5475	833	3709	1027	4131
108	5362	1952	6102	6537	3887
109	245	5688	3545	4569	4194
110	6308	1272	5956	2580	2907
111	111	5175	1462	1838	3639
112	227	5436	6321	2497	438
113	352	275	4729	5600	6784
114	6483	1367	6504	4729	562
115	4612	2363	6892	4658	2392
116	6803	1790	3637	6928	2306
117	1571	4340	4087	5250	3535
118	2230	798	6056	5984	3545
119	4563	3693	2626	140	3557
120	7002	1138	6012	1781	6295
121	1914	6196	1994	1430	1231
122	6324	2929	3786	1480	3387
123	2477	6986	506	4184	2132
124	4397	322	4332	4138	2564
125	2651	4306	1790	2642	3038
126	3033	4418	4993	4751	1514
127	3236	4725	441	3997	1080
128	3056	2912	5973	4949	200
129	3810	1090	1667	3138	880
130	719	3163	4817	4430	3201
131	7063	2030	2522	2901	6196
132	6732	2128	335	2789	405
133	3851	4236	4580	2161	5088
134	4623	4734	1252	6192	4765
135	6731	2380	49	5148	1245
136	1812	5094	6940	4039	3637
137	6054	5704	6815	1003	1409
138	3420	3537	7005	989	7051

139	5785	282	5233	497	5695
140	301	6674	4518	1221	4546
141	3642	4459	6050	592	1238
142	3452	3818	386	6564	5539
143	4793	5706	5564	1670	5888
144	4396	989	6300	2140	6165
145	243	1245	6885	6236	5054
146	5026	3445	2102	2337	104
147	230	3181	5379	2352	1612
148	2274	1157	6553	3299	3121
149	5944	2669	1829	3266	1942
150	3896	1667	5119	401	2282
151	5434	3508	1718	5338	6476
152	4169	4520	544	4928	173
153	5223	4131	3099	5848	6507
154	1275	759	1201	6868	2000
155	2956	7005	3818	5456	4983
156	903	5608	2789	373	1252
157	6882	2459	6280	6525	228
158	1774	1783	3053	4042	5124
159	3578	1825	502	2205	440
160	3242	323	191	232	6659
161	7032	4887	1248	3545	6587
162	5982	7042	4307	6163	1435
163	6417	4242	5583	2457	573
164	1972	6522	4125	1693	3953
165	153	5804	2664	882	7117
166	581	1080	438	3628	6493
167	1995	3387	5139	7045	5119
168	4299	3736	1747	2761	5743
169	3004	405	4544	6246	3383
170	2737	1016	4689	1367	5518
171	6074	2525	1364	3535	6368
172	1324	1643	6716	3388	4202
173	6683	3669	1109	4619	5971
174	2458	4330	1585	5785	7123
175	2576	218	6681	1234	658
176	4263	699	1225	4497	3917
177	6057	3140	6658	5054	5234
178	2949	1110	4242	1708	469
179	5155	1400	3432	506	4094
180	829	5480	5028	1544	5258
181	5797	879	1505	4233	214
182	4250	6110	1043	3942	1693

183	6877	4667	147	1120	4385
184	1495	5518	6144	5434	3665
185	6403	4970	6542	6565	1098
186	1116	1969	1216	1622	2030
187	4749	5876	1132	4292	6591
188	1044	1217	6085	464	1543
189	2072	2821	5867	5713	5966
190	2328	4110	6480	5166	4032
191	4067	171	3469	1887	5936
192	363	2941	4117	5952	5840
193	4345	2031	4328	5300	1894
194	727	4374	2487	3387	1523
195	2670	4343	5450	3926	445
196	347	441	1694	6790	6113
197	4403	6591	4406	4395	1259
198	4514	1472	1148	2929	259
199	763	3698	1102	6167	306
200	807	4025	5695	6293	386

Table S3: Top 200 genes indices selected by different methods from Lung1.

	MIC_{Y-2X}	MIC_{Y-X}	mRMR	RFE	TSG
1	3380	7200	12114	7200	2421
2	3742	7765	11328	5393	5301
3	7393	2421	3333	4336	6139
4	9959	541	718	12308	12114
5	3271	6571	10508	12079	3916
6	7392	8005	2549	12532	11957
7	6618	8180	12196	3334	7046
8	10465	3333	2245	3508	8005
9	2703	8886	3334	2627	718
10	4937	2549	6571	11831	5979
11	8649	3844	2255	3391	8537
12	9925	3250	3902	2255	12152
13	4780	4336	11831	5356	2378
14	5849	11015	11015	3506	3389
15	7028	8370	11292	12277	4083
16	2303	12114	4336	9365	1271
17	7620	5301	9465	2720	1182
18	3384	12200	3844	6164	1246
19	10713	9607	10519	1431	6571
20	6626	869	667	9818	8886
21	7404	3334	3250	11015	7249

22	3887	7249	7625	6584	6629
23	9171	3361	11958	4368	11292
24	3219	12298	6164	8564	2549
25	7796	9707	7849	11828	3337
26	1679	3916	11052	8005	11470
27	4355	11926	11926	9228	633
28	6926	9474	10870	3824	6241
29	8555	11470	1271	9823	5229
30	4371	1246	8005	5337	12153
31	9420	633	9228	11470	3764
32	8854	9863	3202	11328	799
33	12462	1192	1222	3509	3287
34	2736	11958	11841	7795	400
35	10542	3902	12298	541	8842
36	9974	7748	6139	6180	541
37	11402	11957	7765	9043	6734
38	4583	667	12308	9371	3250
39	9439	1271	2624	9299	3383
40	692	8537	11671	7729	9999
41	10909	10519	425	5854	3333
42	4661	6139	7748	2549	11926
43	8996	11841	3912	12215	667
44	2555	9698	8537	3279	5356
45	10835	5979	8370	4480	7765
46	8415	2378	3789	182	12200
47	10563	5806	2244	5835	9312
48	9053	12360	1146	8858	4
49	9079	9824	7200	3278	5751
50	4823	4772	1143	2039	4243
51	8521	2039	4368	7706	8080
52	9672	2122	9607	799	7327
53	12433	2787	3361	1949	2787
54	4375	9823	11830	8178	6600
55	5463	12523	6405	9855	3945
56	6166	12186	9823	1192	7059
57	9015	12254	5301	8172	2882
58	3837	9765	7234	4	5104
59	10966	7046	633	8247	4124
60	4466	11671	12200	9142	10519
61	4493	3383	8180	2331	7200
62	5910	1431	6304	1673	9765
63	7337	11858	9229	2769	333
64	10105	12308	9818	5793	8370
65	10977	6405	12186	12078	7160

66	2583	6821	3861	9452	92
67	5673	3912	2205	399	7678
68	5348	1182	3381	4202	2894
69	11189	5356	8697	1222	3513
70	7299	6185	11470	3844	10386
71	5338	2255	7249	7074	12239
72	2995	11368	7795	6261	3490
73	10177	7722	9707	10219	12186
74	6374	8709	1223	6571	11858
75	8013	5104	2039	6998	11671
76	1248	5847	7000	5958	4335
77	7759	6241	4815	6047	8315
78	6127	7849	2062	9824	4119
79	10362	9843	12407	1085	329
80	2845	12196	869	1612	7991
81	9969	9999	3764	1545	8451
82	5570	11392	3906	11914	2806
83	11541	718	1673	7765	7891
84	9144	3202	2882	1082	11246
85	10919	11955	7891	8179	2907
86	2812	3931	5806	3333	4502
87	12460	5793	11770	2240	7849
88	1206	3764	5847	7140	7453
89	4260	333	646	1611	1024
90	9591	2205	1193	5276	12298
91	6693	8126	3931	598	11770
92	1270	6304	7722	7625	10712
93	8896	4335	2421	7386	630
94	1700	11831	2669	6405	11507
95	8024	11258	8564	9229	11914
96	2967	4083	3508	12298	7396
97	5211	2624	11957	1677	6581
98	1470	92	541	8275	12216
99	7745	2882	2787	1126	3122
100	949	182	8126	522	2624
101	11998	3513	9698	4711	6304
102	1157	1611	12360	11355	8748
103	10997	1673	8172	5323	12242
104	3177	10020	10843	3912	8379
105	9076	12020	1127	6241	7048
106	4338	7160	11368	5330	7748
107	12422	11292	1431	8370	11955
108	4781	3508	4772	12343	3361
109	7855	6734	7080	7048	10471

110	6613	4815	8275	12395	4108
111	9996	3381	1545	4906	6914
112	10910	12464	2735	8537	12464
113	10369	3389	9824	7500	47
114	12197	6164	7069	2150	1301
115	2570	8564	5104	11628	12523
116	5224	10386	9299	4819	9609
117	5055	4424	5356	3211	4821
118	10011	4	9034	2507	5232
119	3347	1612	6094	1254	11485
120	7618	5783	8451	11368	10157
121	3972	8108	8376	6262	8638
122	5851	1782	9772	9785	2122
123	7254	11857	1182	12196	4190
124	3534	7625	7894	3789	3737
125	4456	8393	9758	3166	1227
126	3244	12407	2238	329	9371
127	6595	400	3383	8488	9683
128	10448	2822	9019	12407	9115
129	11340	799	8669	5301	9698
130	3097	34	8393	846	956
131	8398	3490	1192	12210	12306
132	6854	6533	8694	11680	7444
133	9401	6600	6953	9707	9772
134	6644	6733	9683	6270	4319
135	9393	8172	9244	1179	2237
136	9796	5248	6189	11050	9365
137	10063	10471	846	11830	9299
138	8723	5853	8858	10284	6733
139	12515	2806	10386	4348	2822
140	8046	10712	12464	5757	12360
141	10782	8842	10926	6749	9446
142	10363	47	5853	7748	8069
143	12507	6017	9728	7234	3381
144	3149	8080	5393	4174	9351
145	11759	11485	1858	2172	4795
146	2680	4502	9843	3361	6128
147	3355	1136	7160	992	11841
148	8922	9683	4194	12384	9677
149	10904	6094	9863	7327	1464
150	12222	6581	1612	9068	5803
151	11854	9772	10912	3591	4194
152	7445	5835	3455	11841	10805
153	12105	10843	4	1151	9311

154	5524	9857	7046	2721	7007
155	10271	2077	6734	10925	503
156	4912	8909	10174	3202	11958
157	10848	7007	7798	1578	1739
158	4001	4821	1611	5806	5826
159	9759	9229	5854	9489	8765
160	2257	8858	7368	935	5383
161	2946	10290	7700	2234	8065
162	7139	3114	6629	6240	12207
163	7843	7080	647	647	4874
164	2595	11616	3278	1223	8524
165	197	11856	4174	5853	7184
166	8827	12152	3412	11529	11856
167	2513	4368	182	138	3913
168	10597	6576	10722	10640	10020
169	4685	1146	1136	1236	3845
170	10748	9312	5979	7917	3411
171	2125	6748	5796	2748	11857
172	9352	734	343	752	7924
173	7930	3287	11281	6551	102
174	4699	12216	8494	11958	11669
175	10761	12173	11171	7131	2234
176	5408	7069	3287	2521	1027
177	9697	11257	11392	1985	6971
178	545	2669	7678	4090	5051
179	959	10062	1444	521	6821
180	8021	11529	4202	10346	7722
181	3616	8524	10977	8780	1574
182	8724	8843	2221	6691	12138
183	5316	4119	4874	4829	10469
184	5821	5383	12513	6115	11441
185	10315	9535	4228	311	10401
186	10986	10212	3693	7046	6099
187	3650	8275	8108	425	8843
188	4968	11828	748	1782	1192
189	9001	3405	6600	1245	9927
190	11255	6099	12395	12026	10722
191	7879	4174	10925	3287	881
192	10614	7368	5727	6852	5853
193	1118	9660	92	2018	5793
194	9492	8829	11139	5819	9069
195	1691	6667	12429	2244	2148
196	3224	5594	3509	5735	12513
197	3346	3509	4233	4111	5018

198	6482	7074	9535	12306	5949
199	8823	6102	2406	11281	4251
200	9239	6629	10472	4379	1630

Table S4: Top 200 genes selected by MIC(X;Y) from Breast erpos.

No.	Gene Symbol	No.	Gene Symbol	No.	Gene Symbol	No.	Gene Symbol
1	ESR1	51	VGLL1	101	GBP1	151	STK39
2	CA12	52	KIF5C	102	CDKN2A	152	MRPS30
3	CA12	53	CRIP1	103	CPB1	153	ESR1
4	CA12	54	ANXA9	104	FAM174B	154	LPIN1
5	GATA3	55	COX6C	105	ANXA9	155	SALL2
6	AGR2	56	GBP1	106	FOXC1	156	MUC1
7	TBC1D9	57	IL6ST	107	ELF5	157	WWTR1
8	NAT1	58	IGFBP4	108	RABEP1	158	FNDC3B
9	CA12	59	MED13L	109	PRSS23	159	MIA
10	GFRA1	60	KDM4B	110	OGFRL1	160	MIR6872
11	CA12	61	EGFR	111	SLC1A4	161	SFRP1
12	GATA3	62	ELF5	112	CCDC170	162	SERPINB5
13	GATA3	63	DACH1	113	SMCO4	163	UBE2D4
14	DNAJC12	64	ESR1	114	PDZK1	164	C9orf116
15	TFF1	65	MAPT	115	KRT23	165	CXCL12
16	TBC1D9	66	TTC39A	116	NDRG1	166	TRIM2
17	SLC39A6	67	SPDEF	117	FUT8	167	FRAT1
18	SLC39A6	68	GOLM1	118	ALCAM	168	SLC6A14
19	IL6ST	69	TRIM29	119	MAPT	169	GUSBP3
20	TFF3	70	KDM4B	120	ARHGAP35	170	WWP1
21	ABAT	71	CDH3	121	ESR1	171	TTYH1
22	MLPH	72	KRT6B	122	KRT16	172	REPS2
23	FOXA1	73	THBS1	123	CHI3L1	173	SPDEF
24	ERBB4	74	IGF1R	124	AR	174	GJA1
25	MAPT	75	METRN	125	LPIN1	175	LCN2
26	VAV3	76	ROPN1B	126	KCNK15	176	KRT7
27	ADIRF	77	CSRP2	127	VAV3	177	LDLRAD4
28	IL6ST	78	EN1	128	LAMP3	178	BLVRA
29	ABAT	79	AFF3	129	P4HTM	179	TELO2
30	IGF1R	80	DNALI1	130	TCEAL1	180	MIR4680
31	GABRP	81	UGCG	131	HLA-F	181	IKBKB
32	RARRES1	82	RET	132	SEMA3F	182	SERPINE2
33	XBP1	83	CYP2B6	133	SYT1	183	HSD17B4
34	ELOVL2	84	ENPP1	134	BTG3	184	DECR2
35	SYBU	85	C16orf45	135	PRNP	185	GSTP1
36	CELSR1	86	NME3	136	BTG3	186	LAPTM4B
37	C6orf211	87	SCRG1	137	MID1	187	MED13L

38	EVL	88	GREB1	138	TPBG	188	SERPINA5
39	SCUBE2	89	PROM1	139	IRS1	189	PRLR
40	CYP2B6	90	FBP1	140	CIRBP	190	GALNT6
41	NKAIN1	91	MMP7	141	PLAGL1	191	KCNE4
42	FAM171A1	92	WWP1	142	BCAM	192	FZD7
43	RARRES1	93	DACH1	143	PBX1	193	PKP1
44	UGCG	94	NME5	144	LAMB2	194	PNPLA4
45	IL6ST	95	TOX3	145	GPM6B	195	PADI2
46	RHOB	96	RAB26	146	PLAT	196	MCCC2
47	SCCPDH	97	CYP2B7P	147	CLIC4	197	PBX1
48	RARRES1	98	ADCY1	148	ASS1	198	EEF1A2
49	MAGED2	99	KIAA1324	149	C1orf106	199	SLC16A6
50	MYB	100	ZNF552	150	IFRD1	200	SFRP1

Table S5: Top 200 genes selected by MIC(X_1;X_2;Y) from Breast erpos.

No.	Gene Symbol 1	Gene Symbol 2	No.	Gene Symbol 1	Gene Symbol 2
1	SPDEF	TRIT1	51	ESR1	PLAUR
2	LPIN1	BRF2	52	SCCPDH	CREB3L2
3	CEP170	C5orf30	53	SCCPDH	PTPN14
4	CXorf40B	CEP55	54	UGP2	PTP4A2
5	SSH3	KLF11	55	STMN1	BCL2L1
6	CD63	CSTB	56	PBX1	APP
7	SCCPDH	IQCJ-SCHIP1	57	LPIN1	TLE3
8	WWP1	FAM60A	58	MAGED2	AIMP2
9	SSH3	DSC2	59	DBI	RABEP1
10	PAPSS1	ERBB2	60	SCCPDH	CYP7B1
11	PDIA6	KIAA0232	61	PDIA6	PTP4A2
12	AGFG1	MREG	62	CREB3L2	SLC7A8
13	PDIA6	COMMD10	63	CREB3L2	LRBA
14	PTP4A2	ENO1	64	TRIP13	MCCC2
15	ADCY9	TLDC1	65	SHFM1	MAGED2
16	EIF5B	KDM4B	66	MLXIP	KDM4B
17	PRDX3	PSME4	67	CLCN3	RRM2
18	PSME4	CRNKL1	68	COPZ2	SMCO4
19	SSH3	UPF3B	69	IGF1R	TAF11
20	APP	GLUL	70	ANKRA2	FAM60A
21	CYB5R1	TLDC1	71	PTP4A2	AGFG1
22	PDIA6	PTP4A2	72	HMGN1	TSPAN1
23	SEPHS1	CRNKL1	73	ACP1	METRN
24	CLUH	KDM4B	74	SPDEF	NT5DC2
25	BRPF1	SPDEF	75	CDC45	KAT6B
26	PJA2	SERBP1	76	SCCPDH	MAFG
27	PDIA6	MAGED2	77	IL15RA	JADE2

28	EFCAB14	TUBB6	78	EGFR	SLC25A20
29	HMGA1	PTP4A2	79	SIAH2	KIF2C
30	COX8A	HMGA1	80	TUBB3	TSPAN13
31	IGF1R	TTC13	81	SERBP1	PTP4A2
32	SOS1	CXorf40B	82	ESR1	LGALSL
33	HNRNPA1	PSME4	83	AK6	GLG1
34	GPN1	ORMDL2	84	TTLL4	PEX11A
35	PTDSS1	OCEL1	85	PBX1	TLDC1
36	MAGED2	RHEB	86	PTDSS1	PROSC
37	ABCA3	TLDC1	87	KAT6B	MICALL1
38	EGFR	HDAC11	88	TM9SF1	BTG3
39	MED13L	TRIM2	89	PFN2	SPDEF
40	ZNF467	TCF7L1	90	XPC	LPIN1
41	TPX2	GIN1	91	SKP1	PDIA6
42	IGF1R	PTPN14	92	BTG2	STEAP3
43	SCCPDH	MFN1	93	IGF1R	ZNHIT6
44	SCCPDH	NAB1	94	AFF3	RALGDS
45	HMGA1	MZT2A	95	IGF1R	DLX5
46	USP13	SPDEF	96	HMGA1	HMGN2
47	CDC37	KDM4B	97	KIAA0100	YEATS2
48	NCK2	HNRNPDL	98	YWHAQ	PANK3
49	BBS1	TCF7L1	99	CREBL2	NCK2
50	MED13L	HJURP	100	CREBL2	SMCO4

Table S6: Top 200 genes selected by MIC(X;Y) from Breast pcr.

No.	Gene Symbol	No.	Gene Symbol	No.	Gene Symbol	No.	Gene Symbol
1	MAPT	51	TLR5	101	IGKV1OR2-108	151	RARRES1
2	CA12	52	CD58	102	PVALB	152	VSIG4
3	FZD7	53	KLK11	103	PSMD1	153	UNG
4	FOXC1	54	RBKS	104	LBR	154	SLC17A5
5	CA12	55	SFTPB	105	FBL	155	TAPT1
6	TPGS2	56	BEND5	106	TTK	156	GADD45GIP1
7	CBFA2T3	57	N4BP2L2	107	MDK	157	PICALM
8	FABP5	58	EXOG	108	GATA3	158	SCGB1D2
9	BMI1	59	ZNF131	109	ABAT	159	SF3B3
10	GBP1	60	RARRES1	110	SETD1B	160	MZF1
11	BC069756	61	STT3A	111	PKP1	161	GREB1
12	MFGE8	62	MSMB	112	BTG3	162	CYP2B7P
13	GLYR1	63	PRPSAP1	113	NFIB	163	TMOD3
14	ESR1	64	ATP5L	114	MAPK1IP1L	164	RHOB
15	GTPBP8	65	NSDHL	115	FBXW11	165	KCNAB1
16	SRSF7	66	ERBB2	116	SMYD3	166	ALG11
17	MAN1A1	67	SMARCC1	117	HN1L	167	C7orf26

18	ZNF492	68	PDLIM3	118	VPS16	168	HCLS1
19	PGR	69	BAG6	119	TMEM45A	169	NFIB
20	MIR1204	70	PRRC2B	120	GMPS	170	LPCAT4
21	RREB1	71	IGK	121	CBLB	171	RP11-714L20.1
22	GOLGA8A	72	PJA2	122	KCTD3	172	IL6ST
23	PLD1	73	HOXA1	123	DCTN4	173	UNC93A
24	DCTN1	74	IPO9	124	NAT1	174	UPF2
25	BTG3	75	TGFBR2	125	NFIB	175	SPATA31C2
26	ALOXE3	76	TM4SF1	126	TMEM158	176	CENPE
27	RPL5	77	PDSS1	127	SOX4	177	GATA3
28	C4A	78	SRP72	128	MED17	178	EIF4A1
29	HRASLS	79	ERBB4	129	KLRD1	179	IGHG1
30	CEP55	80	NFIB	130	PTP4A2	180	IFNGR1
31	NFIB	81	UBE2L6	131	POU5F1P4	181	CEACAM1
32	GPM6B	82	NPAT	132	TBC1D1	182	TIPARP
33	PLCH2	83	STC1	133	SPRY4	183	LOXL2
34	ADCY9	84	KHDRBS3	134	SAA3P	184	ALDOA
35	NFYA	85	KIAA0141	135	USP2	185	RNF114
36	C18orf32	86	AK021933	136	LILRA1	186	METRN
37	SHB	87	DBT	137	TRPC6	187	PFKP
38	COL8A1	88	SCRG1	138	PPIP5K2	188	CYP2A7
39	SUN1	89	IGHM	139	CACNB3	189	AAGAB
40	IRF5	90	SYNJ1	140	AK022030	190	DESI2
41	ITGB4	91	NUDT6	141	LDB2	191	TIMM17A
42	HMGB1	92	DSTN	142	EEA1	192	NF1
43	IL13RA1	93	CCHCR1	143	WDR11	193	BICD2
44	BID	94	KCNN3	144	NEFL	194	PNPO
45	IDUA	95	AVPI1	145	TMSB15A	195	HPCAL4
46	DESI2	96	RPL10L	146	RTN1	196	CT55
47	JAM2	97	TWSG1	147	GFRA1	197	ZNF750
48	CCNB2	98	SYBU	148	ERBB4	198	ADRA2A
49	LAMB4	99	PLAUR	149	C1GALT1	199	GATA3
50	NCAM1	100	SCUBE2	150	IRF1	200	ZNF44

Table S7: Top 200 genes selected by MIC(X_1;X_2;Y) from Breast pcr.

No.	Gene Symbol 1	Gene Symbol 2	No.	Gene Symbol 1	Gene Symbol 2
1	SLC26A2	PNMT	51	IFI35	COPZ1
2	TP53BP1	RPP40	52	SPINT1	PRR13
3	APOBEC3B	BRMS1	53	ESR1	HJURP
4	ZNF189	SPDEF	54	CYB5A	RBM8A
5	GOT2	RB1CC1	55	HMGCL	AK4
6	SPDEF	LAP3	56	DDX3X	SPDEF
7	PUM2	FAM222B	57	BCL11A	LOC100652930

8	MLF1	ZNF395	58	GORASP2	FAM13B
9	AHNAK2	NAA15	59	CALU	SLTM
10	MIOS	SERHL2	60	COL5A1	TSR3
11	TBC1D9	YTHDF2	61	TSPAN1	VPS54
12	DBI	ANAPC13	62	TMSB15A	SMPD1
13	FIBP	SP110	63	PPP1CB	SAP18
14	SSH3	DNMT1	64	QPRT	GREM1
15	STAG2	SPDEF	65	ACTN1	RABEP2
16	SSH3	PAPD7	66	GPR4	MUC1
17	KDELR2	PJA2	67	CPE	PAIP1
18	DDIT4	GORASP1	68	PCYT2	WNK1
19	EIF1AX	CYB5A	69	CALU	GEMIN2
20	GALNT7	FAM208B	70	CEP152	VGLL1
21	AP1M2	IQCG	71	TEAD4	GEMIN2
22	ENO2	ACACA	72	UBA2	SPDEF
23	SLC2A3	NME3	73	DUSP14	NDUFB6
24	PPP1CB	GATAD1	74	CPE	PNO1
25	EPS8L1	IL17RC	75	GALNT10	ZNF232
26	UBE2D1	LOC101928378	76	B4GALT5	KIAA1324
27	RASGRP3	SERHL2	77	SPDEF	LAP3
28	CELSR2	IL17RB	78	MMP11	SNAPC3
29	THBS1	C6orf106	79	CDK12	DCAF7
30	PLCB4	HFE	80	YIPF2	WNK1
31	CREB3L1	SOBP	81	SSH3	CDK3
32	BIK	ING1	82	IDH3G	ASL
33	SSH3	RSRC2	83	SSH3	TUG1
34	KYNU	SLC6A8	84	DLK1	NFATC1
35	GCHFR	TM4SF1	85	SLC26A2	PAX9
36	CTNNAL1	SPRY4	86	SMURF2	PDC
37	LGALS1	LDOC1	87	JOSD1	IGF1R
38	PLD2	FAM114A1	88	TECR	IFI35
39	WIPF2	RPL22	89	NEO1	SP110
40	MT2A	OAZ1	90	PSMD8	ITPR1
41	GBP1	ASAH1	91	COL1A1	CFLAR
42	TMSB15A	HLA-DOA	92	PNMT	BCL11A
43	SLC25A36	MSL1	93	TES	FBXL5
44	SDC1	MYLK	94	SLC26A2	RALGAPB
45	TOPORS	CYB5A	95	ZBTB20	HRASLS
46	CRCP	GLS2	96	IFI35	LMCD1
47	CIB1	DROSHA	97	PPFIA1	NCAPG
48	CREB3	MFN1	98	RRBP1	CCPG1
49	GYG1	PGAP3	99	DYRK1B	SLC26A2
50	DLK1	KIF4A	100	B4GALT7	RPL39L
